# The effect of topical amiloride eye drops on tear quantity in rabbits

**Published:** 2010-11-04

**Authors:** Shuya Hara, Akihiro Hazama, Masao Miyake, Takashi Kojima, Yasumasa Sasaki, Jun Shimazaki, Murat Dogru, Kazuo Tsubota

**Affiliations:** 1 Social Insurance Chukyo Hospital, Department of Ophthalmology, Nagoya, Japan; 2 Fukushima Medical University, Department of Physiology, Fukushima City, Japan; 3 Keio University School of Medicine, Department of Ophthalmology, Tokyo, Japan; 4 Tokyo Dental College, Department of Ophthalmology, Ichikawa, Japan

## Abstract

**Purpose:**

To investigate the presence of epithelial sodium channels (ENaC) in rabbit and human conjunctival epithelium and to test the effects of topical amiloride, a potassium-sparing diuretic that blocks the ENaC, on tear quantity in rabbits.

**Methods:**

Both healthy normal human and rabbit conjunctival tissues underwent immunohistochemistry staining for ENaC-α and γ subunits as well as for reverse transcription-polymerase chain reaction (RT-PCR) for detection of *ENaC-α* and *ENaC-γ* subunit mRNA expression. Rabbits were instilled topical amiloride eye drops and tear function tests were performed before and after instillations.

**Results:**

Immunohistochemical staining for ENaC-α subunit in all rabbit eyes showed positive staining in apical and basal conjunctival epithelial cells. Human conjunctival epithelia revealed positive staining with ENaC-α antibody especially in the apical and basal layers. Immunohistochemistry staining with ENaC- γ antibody also revealed positive staining of the conjunctival epithelial cells especially in the basal layers. The *ENaC-α* mRNA was detected in samples from healthy white rabbit conjunctival epithelia, and *ENaC-α* and *ENaC- γ* mRNAs were detected in samples from healthy human conjunctival epithelia. The mean tear quantity showed a significant increase at 15 and 30 min compared to the pre-instillation value in eyes assigned to amiloride eye drops. The mean tear quantity at 15 and 30 min was significantly higher in the amiloride group compared to the control eyes.

**Conclusions:**

Topical amiloride application appears to increase the quantity of preocular tears owing to inhibition of conjunctival epithelial sodium channels.

## Introduction

It is well known that the epithelial sodium channels (ENaC) exist in many tissues throughout the body; such channels have been characterized in amphibian skin and urinary bladder, renal collecting duct, distal colon, sweat, and salivary glands, lung, and taste buds. These channels mediate the first step of active Na^+^ reabsorption and play a major role in the maintenance of electrolyte and water homeostasis in all vertebrates [1].

In human ocular tissues, ionic transport has been reported to exist in the corneal endothelium [2] as well as the ciliary body [3], and retinal pigment epithelium [4]. Ionic transport through these tissues is reported to provide the driving force for fluid secretion, a function necessary for the control and maintenance of corneal [5] and lens transparency [6], intraocular pressure [7], and retinal structure [8]. The mucin secreting stratified conjunctival epithelium covers the inner eyelid and bulbar ocular surfaces. Ionic transport by the conjunctival epithelial cells may be important in determining the amount of tears collected in the conjunctival sac which may also affect the stability of the tear film. Although the composition, biochemistry and physiologic aspects of the tear film have been well studied [9-17], the presence and role of sodium channels in human and rabbit conjunctival epithelia and the role of pharmacologically controlled fluid secretion dynamics of the tear film has not been fully investigated and elucidated until now. It is known that the ENaCs are involved with the water resorption in the kidney and that amiloride blocks the ENaC and suppresses the water resorption causing its retention [1].

We initially investigated the presence of ENaC in rabbit and human conjunctival epithelium by performing immunohistochemistry staining and reverse transcription-polymerase chain reaction (RT–PCR) for *ENaC* mRNA expression for the first time in the literature. We also performed animal experiments in rabbits by instilling topical amiloride eye drops to investigate the timewise effects of topical application on tear quantity and to test the feasibility of topical amiloride application as a possible future treatment modality for dry eyes.

## Methods

Seven female Japanese white rabbits (3 kg bodyweight) were obtained from the Shiraishi Experimental Animal Breeding Farm, Tokyo, Japan. One rabbit was anesthetized with 4 ml of intramuscular ketamine and xylazine (1:7 mixture) and topical xylocaine, and conjunctival tissue was excised. All rabbits were handled in strict accordance with the Association for Research in Vision Ophthalmology Statement on the Use of Animals in Ophthalmic and Vision Research. Normal human conjunctival tissues were obtained from the free flap site during pterygium surgery, and informed consent was obtained from all participants. Both human and rabbit conjunctival tissues underwent immunohistochemistry staining for ENaC-α and γ subunits as well as processing for RT–PCR for detection of *ENaC-α* and *ENaC-γ* subunit mRNA expression. Six rabbits were instilled with topical amiloride eye drops and tear function tests were performed before and after instillations.

### Immunohistochemistry

Rabbit and human conjunctival tissues were fixed in 4% paraformaldehyde and dehydrated in ascending grades of alcohol (70%, 80%, 90%, 95%, and 99%: each step once for 1 h, with the last stage in 99% alcohol for 3 h). The samples were then treated in xylene and embedded in paraffin, and 5 μm sections were cut. The sections were treated with xylene three times for 15 min and then treated with 99% ethanol three times for 10 min each. The sections were incubated with 0.3% H_2_O_2_ in methanol for 1 h at room temperature to deactivate internal peroxidase. The step was followed by rehydration in descending grades of alcohol. Then sections were treated with 1% BSA/0.2% gelatin/0.05% saponin/PBS (pH 7.4) three times to block nonspecific signals, and were incubated with primary antibodies overnight at 40 °C. The primary antibodies were goat polyclonal anti-human ENaCs (sc-22239: for ENaC-α, sc-22242: for ENaC-β, and sc-22245: for ENaC-γ; Santa Cruz Biotechnology, Inc., Santa Cruz, CA). The working dilution factors with PBS were 1:2 for rabbit tissue, 1:500 for human ENaC-α, 1:100 for human ENaC-β, and 1:2,500 for human ENaC-γ. Following overnight incubation with primary antibodies, the sections were washed with 0.1% BSA/0.2% gelatin/0.05% saponin/PBS (pH 7.4) three times for 5 min each. Rabbit sections were incubated with Rabbit anti-goat IgG FITC conjugated (F7367; Sigma-Aldrich, St. Louis, MO) diluted 1:50 with PBS for 1 h, then washed three times with PBS. Propidium iodide (1 μg/ml) was used for nucleus staining.

After mounting with Vectorshield (Vector Laboratories, Inc., Burlingame, CA), pictures were obtained with photoimaging system (BX50 and DP70; Olympus Corp., Tokyo, Japan). Human sections were incubated with mouse anti-goat IgG peroxidase conjugated (A9452; Sigma-Aldrich) diluted 1:200 with PBS. Enzymatic staining was performed with 0.2 mg/ml DAB/0.03% H_2_O_2_/Tris-Cl (pH7.5) solution for 10 min followed by counter staining with hematoxylin. Sections were then treated with graded alcohol, xylene, mounted with Eukitt (Sigma-Aldrich) and examined under light microscope. Primary antibodies were excluded in experiments for negative controls.

### RT–PCR for *ENaC* mRNA expression

Total RNA was isolated using RNeasy mini Kit (Qiagen, Inc., Valencia, CA) from 30 mg of homogenized tissues. RNA concentration was quantified by spectrophotometry (Amersham Biosciences AB, Uppsala, Sweden). Two sets of oligonucleotide primers (Thermo Electron Corp., Waltham, MA) were synthesized for RT–PCR analysis as follows: Sense and antisense primers for *ENaC-α* for rabbits were 5′-GAC CTG GAC AGC ATC ACC CAG CAG ACG-3′ and 5′-GCA GCG GGA TGA AGT CAT TCT GCT CTG-3′, for *ENaC-α* for human samples these were 5′-TGG ACT GGA AGG ACT GGA AGA-3′ and 5′-CGG CAG GCG AAG ATG AA-3′, respectively. Sense and antisense primers for beta-actin (*ACTB*) for the rabbit study were 5′-CAA CCG TGA AAA GAT GAC CC-3′ and 5′-CGA TGC CAC AGG ATT CCA TA-3′, and for the human study these were 5′-CGT TGA CAT CCG TAA AGA CCT C-3′ and 5′-GTA CTC CTG CTT GCT GAT CCA C-3′ for the control experiments. The sizes of RT–PCR products were 574 bp for *ENaC-α* for the rabbit and 199 bp for *ENaC-α* for the human study. RT–PCR product sizes were 484 bp for *ACTB* for the rabbit study and 229 bp for *ACTB* for the human study.

The RT–PCR reactions were performed using one-step RT–PCR kit (Qiagen). The amplification program was as follows: 50 °C, 30 min; for reverse transcription, 95 °C, 15 min; for initial Taq enzyme activation, following PCR cycle 94 °C, 30 s; denaturation, 60 °C, 30 s; annealing, 72 °C, 60 s; extension. Five µl of product were run on 3% agarose gel to confirm sizes of PCR products. Optimization for concentration, amplification efficiency, faithful coamplification with housekeeper gene primer and prone sets (glyceraldehyde-3-phosphate dehydrogenase [*GAPDH*]) were also performed.

### Topical amiloride eye drop application experiments

Amiloride eye drops (0.1%) were prepared by dilution of commercially available powder (Wako Pure Chemical Industries, Ltd., Osaka, Japan) with sterile saline solution. Five right rabbit eyes were allocated to amiloride drops, and the left eyes to sterile saline drops. Schirmer test with 10 μl of 0.4% oxybuprocaine topical anesthesia was performed by inserting standard strips (Alcon Laboratories, Inc., Fort Worth, TX) in the lower conjunctival sac for 5 min before as well as 5 min, 15 min, 30 min, and 60 min after amiloride eye drop application. Fifty μl of amiloride eye drops were instilled into the lower conjunctival sac by a micropipette to the right eyes and 50 μl of sterile saline was instilled to the left eyes at the beginning of the experiments.

Rabbit conjunctival tissues were excised after amiloride drop application, stained with hematoxylin-eosin and examined under light microscopy to investigate any morphological alterations due to eye drop application.

### Corneal fluorescein staining

Two μl of 1% fluorescein solution were applied by micropipette before amiloride application and at 60 min after to check the effects of the topical amiloride application on the corneal epithelium. The fluorescein staining was scored according to the protocol described by Shimmura et al. [18-20]. Briefly, the cornea was divided into three equal areas of upper, middle and inferior corneal compartments. Each compartment was graded on a scale of 0 (no staining) to 3 points (intense staining). A fluorescein staining score above 1 point was considered abnormal (maximum: 9 points). The cornea was inspected with a hand held slit lamp microscopy (Carl Zeiss AG, Oberkochen, Germany).

## Results

### Immunohistochemical confirmation of ENaC-α, β, and γ subunits in rabbit conjunctiva

**Figure 1:**
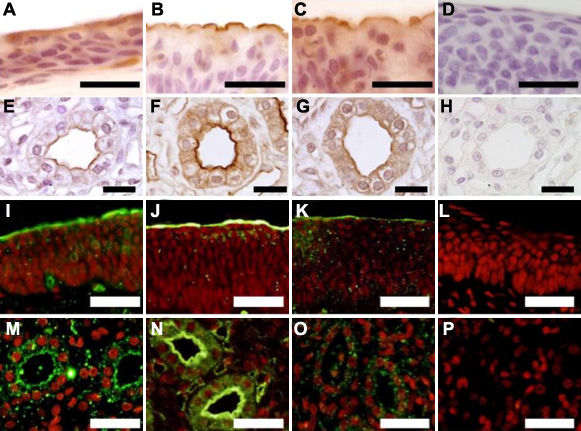
Expression of ENaC subunits in rabbit conjunctiva in immunohistochemical stainnings. ENaC-α (**A** and **I**), ENaC-β (**B** and **J**), and ENaC-γ (**C** and **K**) were positive at apical layer of conjunctiva. As a positive control, ENaC-α (**E** and **M**), ENaC-β (**F** and **N**), and ENaC-γ (**G** and **O**) were positive at inner medullas of kidney. Isotype control specimen showed no positive staining in conjunctiva (**D** and **L**) and kidney (**H** and **P**). Scale bars in **A**-**H** represent 20 μm and scale bars in **I**-**P** represent 40 μm.

Immunohistochemical staining for ENaC-α, β, and γ subunits in all rabbit eyes showed positive staining in apical and basal conjunctival epithelial cells as well as in inner medullas of kidney for positive control as shown in Figure 1.

### RT–PCR for *ENaC-α* mRNA expression in rabbit conjunctiva

**Figure 2:**
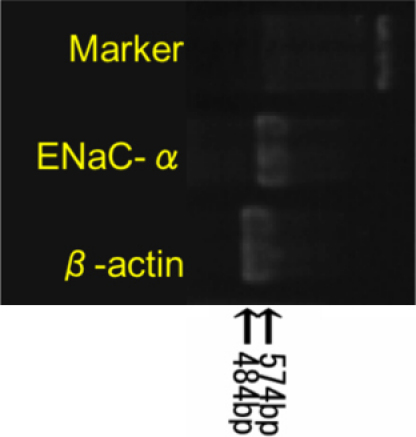
Expression of *ENaC-α* and housekeeping gene (*ACTB*) in rabbit conjunctiva.

To assess the expression of *ENaC-α* in rabbit conjunctival epithelial cells, RT–PCR technique using primer for *ENaC-α* was adopted. MRNA of *ENaC-α* was detected in samples from healthy white rabbit conjunctival epithelia (Figure 2). Because mRNAs of the same samples from experimentally manipulated conjunctiva were amplified by primers of *ATCB* (Figure 2), we verified that these samples were suitable for detection of the mRNA of interest.

### Immunohistochemical confirmation of ENaC-α and ENaC-γ subunits in human conjunctiva

**Figure 3:**
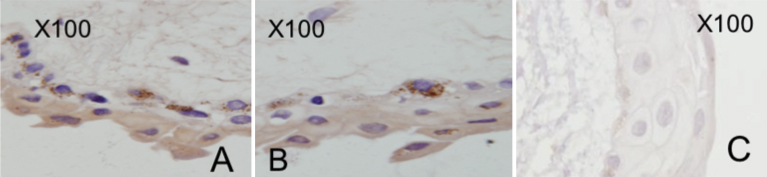
Immunohistochemistry stainings of ENaC- α and ENaC-γ subunits in human conjunctiva.

Human conjunctival epithelium revealed positive staining with ENaC-α antibody especially in the apical and basal layers (Figure 3A). Immunohistochemistry staining with ENaC-γ antibody also revealed positive staining of the conjunctival epithelial cells especially in the basal layers with some staining in the apical layers (Figure 3B). Isotype control specimens did not reveal any staining with the relevant antibodies. A representative isotype control specimen for human conjunctival ENaC-α immunohistochemical staining is shown in Figure 3C.

### RT–PCR for *ENaC-α* and *ENaC-γ* mRNA expression in human conjunctiva

**Figure 4:**
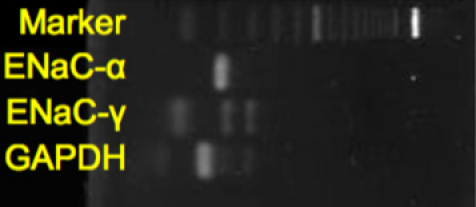
Expression of *CIC-2*, *ENaC-α*, *ENaC-γ*, and housekeeping gene (*ACTB*) in human conjunctiva.

To assess the expression of *ENaC-α* and *ENaC-γ* in human conjunctival epithelial cells, RT–PCR technique using primers for *ENaC-α* and *ENaC- γ* was adopted. MRNA of *ENaC-α* and *ENaC- γ* was detected in samples from healthy human conjunctival epithelia obtained from part of the flap site from surgery for pterygium (Figure 4). Because mRNAs of the same samples from experimentally manipulated conjunctiva were amplified by primers of *GAPDH* (Figure 4, lower band), we verified that these samples were suitable for detection of the mRNA of interest.

### The change of Schirmer test value with topical amiloride eye drops

**Figure 5:**
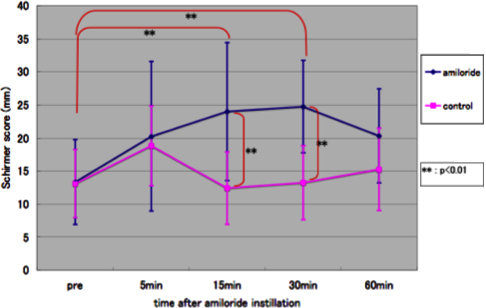
The change of Schirmer test value in topical amiloride eye drop experiment.

The mean tear quantity showed a significant increase at 15 and 30 min compared to the pre-instillation value in eyes assigned to amiloride eye drops. The mean tear quantity at 15 and 30 min was significantly higher in the amiloride group compared to control eyes (Figure 5).

### Slit lamp examination and fluorescein staining

Slit lamp examinations did not show any conjunctival and corneal injection, edema, or sign of inflammation before and after eye drop instillations. Likewise positive corneal fluorescein staining was not detected before or at the end of topical eye drop instillations during the experiments. Hematoxylin-eosin staining of rabbit conjunctival and corneal tissue specimens at the end of amiloride eye drop instillations did not reveal any gross morphological alterations (data not shown).

## Discussion

Current conventional treatment for dry eye mainly consists of artificial tear instillations and punctum plug insertion with the aim to increase the preocular tear volume [21-23]. Recently, newer muscarinic or P2Y2 receptor agonists such as peroral cevimelin [24] and diquasofol tetrasodium, which increase the preocular tear quantity by stimulating the lacrimal gland, have been reported to be effective in the treatment of severe dry eyes. Agents which are among the ophthalmologist’s armamentarium in the treatment of dry eye syndrome include DE-089, and 15-S-HETE which principally act by increasing the ocular surface mucin secretion and improving the tear stability [25]. Because the conjunctiva is a “fluid secreting” epithelium [26], we considered the idea of applying topical amiloride eye drops, which we hoped would increase the preocular tear quantity through inhibition of conjunctival epithelial sodium channels. We believed that such an inhibition would result in suppression of the absorption of the “existing preocular tears” by the ocular surface epithelium, resulting in increased availability of one’s own tear secretion to the ocular surface. Before we tested this hypothesis, we performed immunohistochemical and RT–PCR experiments to confirm the existence of epithelial sodium channels in rabbit and human conjunctival tissues.

The existence of ENaC in the rat conjunctival epithelium has already been reported [27]. Kompella et al. [28,29] confirmed the existence of sodium epithelial channels in rabbit conjunctiva with ionic transport experiments. However, they had not performed immunohistochemistry or RT–PCR for confirmation of the presence of ENaC. The presence of sodium channels in the rabbit conjunctiva or in humans has not been investigated by immunohistochemistry so far to our knowledge.

In this study, we confirmed the presence of ENaC in rabbit and human conjunctival epithelia using renal epithelium as the positive control. For the next step, we performed topical amiloride eye drop instillations in rabbits and investigated the timewise alterations in tear quantity. The preocular tear quantity was observed to increase for the first 5 min, which might have, in part resulted from the instillation of the eye drops.

Interestingly, we noted that although the preocular tear quantity continued to increase afterward in the eyes that were allocated to amiloride eye drops, the contralateral eyes assigned to saline drops showed a significant decrease in tear quantity, probably due to the drainage of the preocular tears from the lacrimal puncta. It is our belief that the significantly higher tear volumes at 15 and 30 min may be due to accumulation of available tears at a level exceeding the drainage of tears due to amiloride effects on the ocular surface epithelia.

Data from Watsky et al. [30] previously suggested that the rabbit conjunctival surface area was 9 times larger than the corneal surface area and that the conjunctiva may play a dominant role in regulating electrolyte and fluid balance in the tear film. In our observations, amiloride eye drops caused a timewise increase in tear quantity as assessed by the Schirmer test, and the effect of amiloride eye drops on the preocular tear quantity was maintained for 30 min which started to decline afterward. It would be interesting to test the dose-dependent effects of amiloride eye drops and concurrent tear electrolyte changes in animal dry eye models in future experiments. Whether inhibition of ENaC by amiloride which leads to reduced absorption of tears by the conjunctival epithelium induces functional consequences on the conjunctival epithelium itself or not also deserves further investigation. Indeed, such inhibition may affect the intracellular and extracellular water ratio and result in cellular dehydration. Further studies employing bioimpedance analyses methods to test the conjunctival cellular hydration with amiloride application would provide very interesting information [31].

Slit lamp examinations and ultrastructural observations did not reveal corneal staining, crack lines or allergic reactions or any related morphological alterations in the light microscope (data not shown) as adverse effects of topical amiloride application. However, we believe that this claim should be further tested with electron microscopical observations and with more frequent instillations of amiloride eye drops in animal models.

Safety experiments should also assess time-wise changes in corneal endothelial ultrastructural morphology and counts, and intraocular pressure measurements since sodium channels exist in the pigmented ciliary body epithelium and corneal endothelium. In summary, the preliminary findings of the current study confirmed the presence of sodium channels in rabbit and human conjunctival epithelia and that topical amiloride application may be increasing the quantity of preocular tears due to inhibition of these channels. The preliminary findings from this study justify further experimental research on amiloride as a possible treatment alternative for dry eye disease.
